# Systematic review of conservation interventions to promote voluntary behavior change

**DOI:** 10.1111/cobi.14000

**Published:** 2022-12-08

**Authors:** Laura Thomas‐Walters, Jamie McCallum, Ryan Montgomery, Claire Petros, Anita K. Y. Wan, Diogo Veríssimo

**Affiliations:** ^1^ Oregon State University Corvallis Oregon USA; ^2^ Force for Nature Comber UK; ^3^ Independent Researcher UK; ^4^ University of Oxford Oxford UK; ^5^ Sun Yat‐Sen University Guangzhou China

**Keywords:** behavior change, human behavior, nudging, OHAT, social marketing, social science, cambio conductual, ciencias sociales, comportamiento humano, estimulación, marketing social, OETS, cambio conductual, ciencias sociales, comportamiento humano, estimulación, marketing social, OETS, 行为改变, 人类行为, 说服, 健康评估和转化办公室(OHAT), 社会营销, 社会科学

## Abstract

Understanding human behavior is vital to developing interventions that effectively lead to proenvironmental behavior change, whether the focus is at the individual or societal level. However, interventions in many fields have historically lacked robust forms of evaluation, which makes it hard to be confident that these conservation interventions have successfully helped protect the environment. We conducted a systematic review to assess how effective nonpecuniary and nonregulatory interventions have been in changing environmental behavior. We applied the Office of Health Assessment and Translation systematic review methodology. We started with more than 300,000 papers and reports returned by our search terms and after critical appraisal of quality identified 128 individual studies that merited inclusion in the review. We classified interventions by thematic area, type of intervention, the number of times audiences were exposed to interventions, and the length of time interventions ran. Most studies reported a positive effect (*n* = 96). The next most common outcome was no effect (*n* = 28). Few studies reported negative (*n* = 1) or mixed (*n* = 3) effects. Education, prompts, and feedback interventions resulted in positive behavior change. Combining multiple interventions was the most effective. Neither exposure duration nor frequency affected the likelihood of desired behavioral change. Comparatively few studies tested the effects of voluntary interventions on non‐Western populations (*n* = 17) or measured actual ecological outcome behavior (*n* = 1). Similarly, few studies examined conservation devices (e.g., energy‐efficient stoves) (*n* = 9) and demonstrations (e.g., modeling the desired behavior) (*n* = 5). There is a clear need to both improve the quality of the impact evaluation conducted and the reporting standards for intervention results.

## Resumen

Es vital entender el comportamiento humano para desarrollar intervenciones que lleven efectivamente a un cambio proambiental en el comportamiento, ya sea que estén enfocadas a nivel individual o de sociedad. Sin embargo, históricamente las intervenciones han carecido de evaluaciones sólidas en muchas áreas, lo que dificulta confiar en que estas intervenciones de conservación hayan ayudado exitosamente a proteger el ambiente. Realizamos una revisión sistemática para analizar cuán efectivas han sido las intervenciones no pecuniarias y no regulatorias en el cambio del comportamiento ambiental. Aplicamos la metodología de revisión sistemática de la Oficina de Evaluación y Traducción de la Salud (OETS). Comenzamos con más de 300,000 artículos y reportes arrojados por nuestros términos de búsqueda y después de una valoración crítica de calidad, identificamos 128 estudios individuales que merecían su inclusión dentro de la revisión. Clasificamos las intervenciones en área temática, tipo de intervención, número de veces que el público estuvo expuesto a las intervenciones y duración de las intervenciones. La mayoría de los estudios reportaron un efecto positivo (*n* = 96). El siguiente resultado más común fue sin efecto (*n* = 28). Pocos estudios reportaron efectos negativos (*n* = 1) o mixtos (*n* = 3). Las intervenciones educativas, de indicaciones y de retroalimentación resultaron en un cambio conductual positivo. La combinación de las múltiples intervenciones fue la más efectiva. Ni la duración de la exposición ni la frecuencia afectaron la probabilidad del cambio conductual deseado. En comparación, pocos estudios analizaron los efectos de las intervenciones voluntarias sobre las poblaciones no occidentales (*n* = 17) o midieron el comportamiento ecológico resultante actual (*n* = 1). De manera similar, pocos estudios examinaron los dispositivos (p. ej.: estufas ahorradoras de energía) (*n* = 9) y demostraciones (p. ej.: modelar un comportamiento deseado) (*n* = 5) de conservación. Hay una necesidad evidente de mejorar la calidad de las evaluaciones de impacto realizadas y de reportar los estándares para los resultados de las intervenciones.

## 【摘要】

对人类行为的理解对于制定有效促进环保行为改变的干预措施至关重要, 无论其重点是在个人还是社会层面。然而, 许多领域的干预措施历来缺乏稳健的评估方法, 以致人们很难确信这些保护干预措施成功促进了环境保护。本研究通过系统综述评估了金钱和监管以外的干预措施在环保行为改变方面的有效性。我们应用了美国国立环境健康科学研究所国家毒理学项目健康评估和转化办公室的系统综述方法。研究者对搜索得到的30多万篇论文和报告进行了严格的质量评估, 确定了128项纳入综述的独立研究。接下来, 我们按照主题领域、干预类型、对受众进行干预的次数和干预时长对干预进行了分类。结果显示, 大多数研究都报告了积极的干预效果(*n*=96), 其次是没有效果(*n*=28), 很少有研究报道出现负面效果(*n*=1)或混合效果(*n*=3)。教育、提示和反馈干预都可以引起积极的行为改变, 而多种干预措施相结合则是最有效的。此外, 接触时间和频率都不影响预期行为改变的可能性。相对而言, 很少有研究检验自愿干预对非西方人口的影响(*n*=17)或检验实际的生态学行为结果(*n*=1)。同样地, 也很少有研究关注保护设备(如节能炉)(*n*=9)和示范(如模拟预期行为)(*n*=5)。我们提出, 未来的研究既需要提高影响评估的质量, 也需要提高干预结果的报告标准。**【翻译:胡怡思;审校:聂永刚】**

## INTRODUCTION

Humanity is having ever greater impacts on the environment, and those impacts are driven by human decision‐making (Lewis & Maslin, 2015). Many people lead unsustainable lifestyles, particularly in higher‐income countries, which contributes to major environmental problems, such as climate change and biodiversity loss (Cowling, 2014; Fischer et al., 2012). Problematic behaviors, such as excessive water and energy use, need to be addressed urgently (OECD, 2011). Accordingly, conservation as a discipline has increasingly embraced the social sciences to aid in the design and evaluation of behavior change interventions (Bennett et al., 2017; Moon et al., 2019). Understanding human behavior is vital to developing interventions that mitigate threats to the environment and effectively lead to proenvironmental behavior change, whether the focus is at the individual or societal level. However, these interventions have historically lacked robust evaluation, which makes it hard to know whether and how conservation interventions have helped protect the natural world (Curzon & Kontoleon, 2016; Josefsson et al., 2020; Junker et al., 2020).

Traditional responses to the environmental crisis have been mainly policy‐based (Lucas et al., 2008; Science and Technology Select Committee, 2011). Central among them have been legislation that eliminates or restricts choice and fiscal incentives or disincentives (Lucas et al., 2008; Taylor et al., 2013). For example, governments have implemented restrictions on the disposal of waste and charge for single‐use carrier bags (Goodstein & Polasky, 2014; Poortinga et al., 2013). Although important, these policies can be resource‐intensive and require political will to implement (Allcott, 2011; Schubert, 2017). They may be politically unpopular as they are intrusive and involve the loss of liberty (although the restriction of environmental harm may benefit the liberty of people in society more widely [Science and Technology Select Committee, 2011]). Pecuniary interventions also require consistent funding in the long term to be sustainable and raise questions around autonomy and power, especially in socioeconomically disadvantaged groups (Marteau et al., 2009). Noncoercive approaches to behavior change have received increasing interest because people retain the freedom to make the choices they wish without concern for legal or financial repercussions, and reliance on political will is lessened (Greenfield & Veríssimo, 2018; Schubert, 2017).

Research testing the effectiveness of these approaches has been conducted for decades (e.g., Asch & Shore, 1975; Krauss et al., 1978), but there still is not a cohesive body of evidence to guide policy makers and practitioners. This could be due to publication in multiple disciplines, including but not limited to social marketing, environmental education, and behavioral economics (Veríssimo & Wan, 2018; Hungerford & Volk, 1990; Lehner et al., 2016). Syntheses of voluntary interventions in the environmental field exist, but they tend to be narrative reviews; include only specific evidence types, such as randomized control trials (RCTs); focus on a select thematic area, such as energy consumption; or test a specific intervention type, such as education (e.g., Abrahamse et al., 2005; Abrahamse & Steg, 2013, Byerly et al., 2018; Heimlich & Ardoin, 2008; Nisa et al., 2019; Schubert, 2017; Wolske et al., 2020; Wynes et al., 2018). Reviews also often include proxies for behavior change, such as changes in behavioral intentions or attitudes. Although these proxies have a role in research, their correlation with behavior is not strong. For example, results of a meta‐analysis show that intentions account for only 28% of the variance in prospective measures of behavior (Sheeran, 2002). In this systematic review, we focused on actual behaviors with clear environmental impacts and incorporated a broader range of experimental designs and intervention types, and had a broader scope that explicitly included the gray literature. We also included a rigorous quality assessment process to ensure only robust methodologies are part of the final synthesis.

Systematic reviews synthesize a body of evidence to explore specific research questions. They are the most reliable and comprehensive statement about what works and provide useful information for policy makers and practitioners (Johnson & Hennessy, 2019; Munn et al., 2018). The transparency and rigor of systematic reviews can be enhanced by following a set of accepted principles, such as the Cochrane and Campbell Collaboration (2013) standards or the Office of Health Assessment and Translation (OHAT) framework (Rooney et al., 2014).

The OHAT approach is a systematic review methodology that increases transparency, consistency, and efficiency in summarizing environmental health‐based findings, with the additional goal of improving data management and display (OHAT 2014). It draws on the best public health protocols (e.g., PRISMA, PECOTS, and Campbell Collaboration) while being able to cope with the broader set of conditions and wide range of data types required in the wider environmental health sciences. For example, it allows for the inclusion of relevant and high‐quality papers in the gray literature to help minimize publication bias (Savoie et al., 2003). It also embraces experimental designs beyond RCTs, an important factor for environmental reviews. It is not always feasible or appropriate to perform an RCT, and in some areas (such as biodiversity conservation), there are very few to learn from. Including only these designs, therefore, excludes a large body of evidence (Christie, 2020). Moreover, there are a variety of alternative, rigorous, quasi‐experimental designs that apply techniques, such as matching, synthetic control, or regression discontinuity to control for observed and unobserved covariates, that are comparable in levels of bias to RCTs (Christie et al., 2019; Pynegar et al., 2021). An RCT is also vulnerable to biases linked, for example, to randomization failure or differential attrition (Jadad & Enkin, 2007). Unless there are other risk factors (e.g., see step 5 in the Methods), rigorous quasi‐experimental designs can be treated with a similar level of confidence to RCTs.

In our systematic review, we explored how effective nonpecuniary and nonregulatory interventions have been in changing environmental behaviors. To do so, we included only studies measuring actual behavior. In particular, we focused on the quality and rigor of the evidence base. We examined the strength of evidence that different types of interventions, such as feedback or goal setting, will result in desired behavior change. We also identified important gaps in the literature.

## METHODS

**FIGURE 1:**
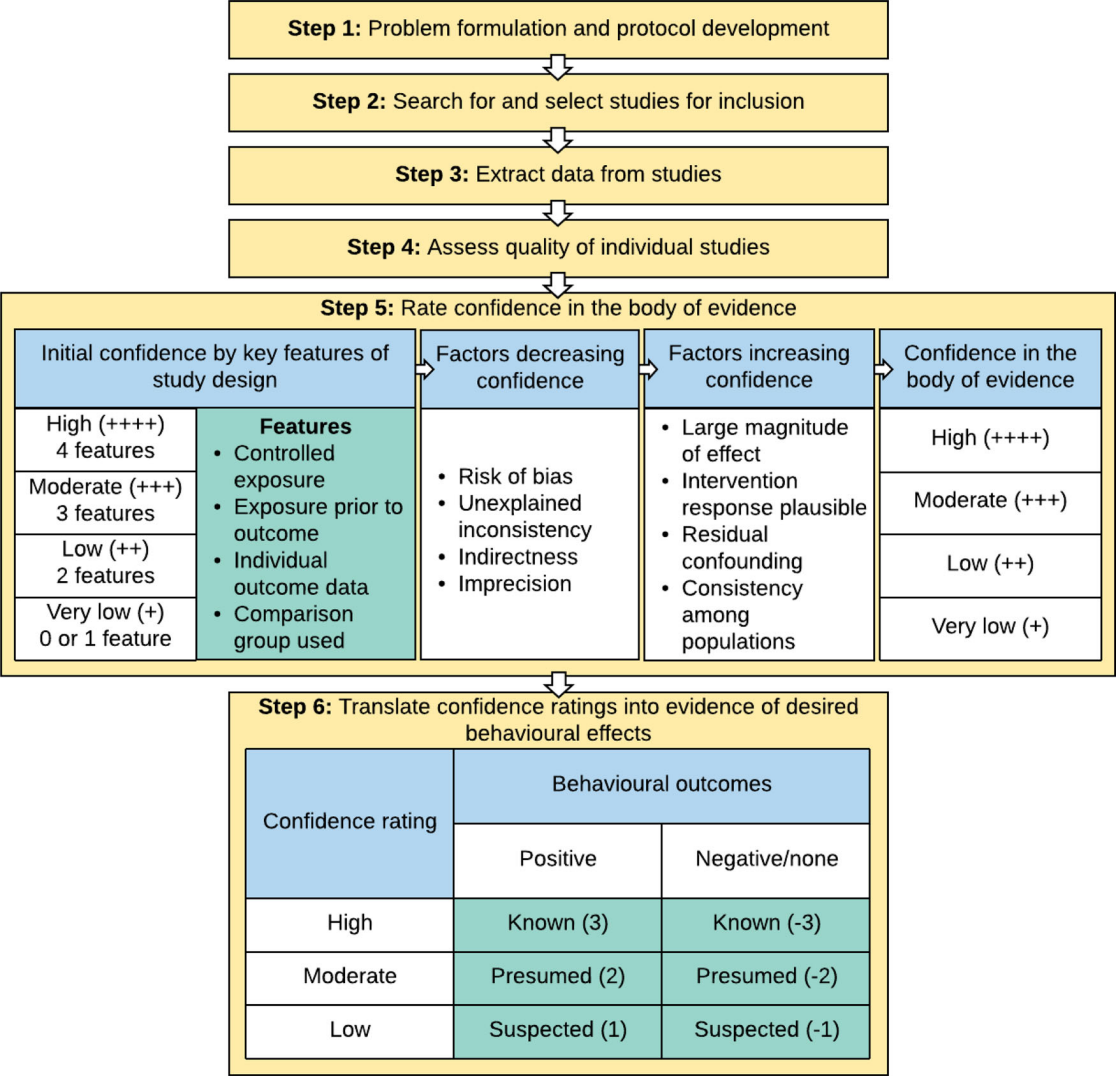
Adaptation of Office of Health Assessment and Translation (2014) systematic literature review protocol to a systematic review of studies evaluating how effective nonpecuniary and nonregulatory interventions have been in changing environmental behaviors

We adapted the OHAT 7‐step framework for systematic reviews (Akers et al., 2009; Rooney et al., 2014) (Figure 1). (We excluded OHAT's final step, which is combining evidence streams for human studies and animal studies, because it was not applicable to our review.) The breadth and inclusive nature of the process enabled us to create some degree of standardization across studies that varied in experimental design and outcome measurement. We sent the review protocol that we developed to two external experts for feedback before the start of the study. As a result of this feedback, minor changes to the protocol were made, including the addition of keywords and clarification of the scope of the review.

### Problem formulation and protocol development (step 1)

We wanted to cast a wide net to include all nonregulatory and nonpecuniary interventions that tried to solve an environmental threat by changing human behavior. Studies may have been published in fields as distinct as waste management, sustainable transport, or social marketing. We specifically focused on assessing the quality of the evidence base and identifying where there were gaps. We were also interested in direct behavioral measures, not just proxies, such as attitudes, intentions, or self‐reported behavior. Outcomes were behaviors with a clear environmental impact, such as water or energy consumption, travel mode choice (e.g., public transport vs. car journeys), recycling participation (frequency and volume), or littering.

### Literature search, processing, and selection (step 2)

From February 26 to June 5, 2015, we systematically searched multiple databases to identify high‐quality and relevant studies from the peer‐reviewed and gray literature. We developed our keyword search strategy based on electronic searches of bibliographic databases or platforms, project funding databases, and specialized internet search engines and repositories (summary in Table 1, full details in Appendix S1). The selected search terms provided broad coverage of environmental behaviors and approaches to behavior change, while keeping the focus on actual behaviors. No language or date restrictions were applied to the searches, although only English keywords were used. The ISI Web of Science and EBSCOhost are platforms that provide access to a wide range of bibliographic databases, and the specific databases we accessed are listed in Appendix S2. Other electronic databases and indexes for peer‐reviewed literature included: SciVerse's Scopus, International Bibliography of the Social Sciences, PsycINFO, Google Scholar, Education Resources Information Center, Environmental Evidence, Campbell Collaboration systematic review database, and International Initiative for Impact Evaluation review database.

**TABLE 1:** Search terms used in the comprehensive literature search for studies evaluating how effective nonpecuniary and nonregulatory interventions have been in changing environmental behaviors

“education” AND OR “conservation” OR “outdoor” OR “ecology” OR “adventure” OR “global” OR “field studies”
“social marketing” AND “conservation” OR “biodiversity” OR “species” OR “habitat” OR “wildlife” OR “nature” OR “environment”
“community based conservation” OR “community‐based conservation”
“integrated conservation and development”
“community conservation”
“community based natural resource management” OR “community‐based natural resource management”
“energy conservation” AND “behavior change”
“water conservation” AND “behavior change”
“recycling” AND “behavior change”
“littering” AND “behavior change”
“source reduction” AND “waste” AND “behavior change”
“reducing consumption” AND “behavior change”
“composting” AND “behavior change”
“carpooling” AND “behavior change”
“fuel efficient vehicles” AND “behavior change”
“walking” AND “behavior change”
“mass transit” AND “behavior change”
“biking” AND “behavior change”
“volunteering” AND “behavior”

For gray literature, we also searched ProQuest Digital Dissertations and Theses, Policy File, Environmental Education Resource Assistant, Canadian Evaluation Society Unpublished Literature Bank, System for Information on Grey Literature in Europe, CORDIS Library, Fostering Sustainable Behavior: Community Based Social Marketing, Tools of Change, Rufford Foundation project database, Conservation Leadership Award project database, Rainforest Alliance Eco‐Index, and Darwin Initiative Project Database.

Boolean operators (i.e., AND and OR) were used as appropriate. For each database, the number of hits per search phrase in titles and abstracts was recorded. The number of records retrieved for the largest bibliographic databases and platforms is listed in Appendix S3. There were additional searches for British and American English spellings of keywords (e.g., *behaviour* and *behavior*). All searches were noted and tracked on a Microsoft Excel spreadsheet for reference.

All records, including the full texts, were screened manually by two coauthors, A.K.Y.W. and D.V., who split the workload in half. Duplicates in the initial database of records were automatically identified through EndNote and then confirmed manually. We triaged the studies in three stages: title, abstract, and full‐text review, and included only empirical, primary data studies. At each stage, studies were categorized as accept, maybe, or reject. This was based on the explicit inclusion or exclusion criteria in Table 2. Two reviewers independently tested the reliability of the triage process with 100 random records. We calculated agreement with Cohen's kappa coefficient; the cutoff for a substantial agreement was >0.6 (McHugh, 2012).

**TABLE 2:** Inclusion and exclusion criteria used to determine study eligibility in a review of studies evaluating how effective nonpecuniary and nonregulatory interventions have been in changing environmental behaviors

PECO	Inclusion criteria
Population	no age or geographic restrictions
Exposure	intervention not pecuniary or regulatory
Comparator	includes a control
	control must be independent
	rationale given detailing why control is comparable to treatment
Outcome	behavioral outcome (i.e., not just knowledge, attitudes, or norms)
	behavioral outcome relevant to the environment

Abbreviation: PECO = population, exposure, comparison, and outcomes.

### Extract data from studies (step 3)

Once we had a complete data set of eligible studies, we extracted data for each record (Appendix S4). All studies were listed with administrative identifiers, including record source, title, first author, and publication year. We categorized them into one of six themes from the Community‐Based Social Marketing classification (McKenzie‐Mohr & Schultz, 2014 [https://cbsm.com/]) of agriculture and conservation, energy, transportation, water, waste, and pollution, or a mix of multiple themes. As well as PECOTS (population, exposure, comparison, outcomes, and time), further information on the intervention context, design, and measure of outcomes were retrieved for each article to obtain data for an overall understanding of evaluation measures undertaken by project organizers.

We also developed a taxonomy of different intervention types. We drew on existing behavioral intervention taxonomies (Abraham & Michie, 2008; Dolan et al., 2012; Kok et al., 2016; Michie et al., 2015; Michie et al., 2013), but tailored it to the types of interventions present in the review studies. There were six main types: education, demonstrations, conservation devices, feedback, goal setting, and prompts (Table 3).

**TABLE 3:** Taxonomy of intervention types in studies evaluating how effective nonpecuniary and nonregulatory interventions have been in changing environmental behaviors

Intervention type	Definition	Example
Education	involves imparting information to increase knowledge or understanding of a behavior or issue	visiting households to discuss the benefits of recycling and local recycling service (Cotterill et al., 2009)
Demonstrations	model a desired behavior, enabling audiences to learn by observation	conspicuously disposing of food waste in appropriate receptacle in a restaurant (Sussman & Gifford, 2013)
Conservation devices	facilitate the performance of a desired behavior with new technologies or improved services	supplying more energy‐efficient stoves to reduce fuelwood consumption (Yin, 2013)
Feedback	provide data on personal behavior, possibly with comparison to a stated goal or behavior of others	home energy reports featuring personalized energy use feedback (Allcott & Rogers, 2014)
Goal setting	encourage audiences to commit to an explicit behavioral target	asking energy consumers to set a specific energy‐saving goal (Loock et al., 2013)
Prompts	uses environmental or social cues to remind audiences to perform a behavior	displaying signs with persuasive messages to remind tourists to pick up litter (Brown et al., 2010)

### Initial quality assessment of individual studies (step 4)

Following OHAT guidelines, we ranked each study based on the quality of reporting, relevance of experimental design to an outcome, and risk of bias (Rooney et al., 2014). Quality of reporting refers to how well a study was completed or reported. Relevance refers to the relevance of experimental design to the behavioral outcome. Risk of bias includes external validity or directness and applicability (i.e., how well a study addresses the topic under review). The first two criteria were rated from 1 (low) to 3 (high), and the risk of bias was rated from 1 (definitely high) to 4 (definitely low). We then added the three ranks for a total score out of 10. Studies that scored <6 were removed from the review because they were problematic in multiple key aspects of study quality (Office of Health Assessment and Translation, 2015).

### Confidence rating for studies (step 5)

**FIGURE 2:**
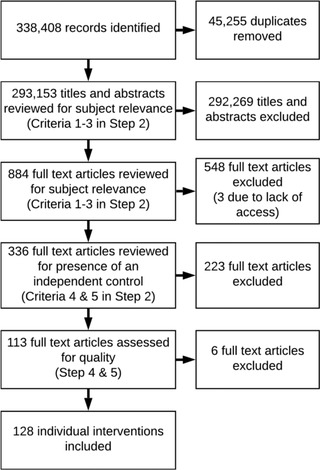
Process used to select articles in a systematic review of studies evaluating how effective nonpecuniary and nonregulatory interventions have been in changing environmental behaviors

At this stage in the OHAT protocol, similar studies would be clumped together to enable the processing of a large number of papers and to determine common threads. However, we did not do this. There are multiple ways we could have categorized the studies (e.g., thematic and intervention types). Because a relatively small number of papers made it to this stage (Figure 2) and there was considerable heterogeneity in experimental design and behavioral outcomes, we rated each study individually.

We developed a confidence rating for each study based on the presence or absence of key features identified in the OHAT process (Schünemann et al., 2011). Studies earned 1 point for each of the following eight features that would increase our confidence in the study result. First, exposure to the intervention was controlled by researchers. This allowed us to largely eliminate confounding through randomization of the allocation of exposure. Second, exposure to the intervention occurred prior to the measurement of outcomes. Third, outcome measurements were collected at the individual level. Fourth, a comparison or control group was used. Fifth, studies that showed >50% magnitude of effect relative to the control group within the population. Sixth, if there was a degree of plausibility between the level of exposure and outcome, then it was more likely the result did not occur due to chance. Whether the degree of change in a population is subject to the degree of a given exposure is especially relevant when looking at studies that varied degrees of the same exposure or used factorial study design when multiple exposures were applied in different combinations. Seventh, residual confounding, which refers to effect modification that would bias the effect estimate toward the null. If an effect or association is reported despite the presence of residual confounding that would diminish the association; confidence in the association increases. Eighth, consistency among control and treatment populations, which refers to the extreme similarity in a population, notably the robustness of the replicates and comparable controls, intervention, and treatment.

The presence of any of the following four features that would decrease our confidence in the study result meant a 1‐point deduction. First, the risk of bias was extrapolated from the OHAT step 4 risk‐of‐bias score. Any study which rated probably high or definitely high qualified for the point deduction. Second, unexplained inconsistency referred to the external validity or indirect measures of the behavioral outcome, which was obtained by reading the results and discussion. Third, indirectness was assessed using the relevance scores from the OHAT step 4, for which low relevance qualified for the point deduction. Fourth, imprecision was the degree of certainty surrounding an effect estimate and was assessed based on sample size, power of the statistical methods used, and confidence intervals (e.g., standard deviation greater than the mean or an odds ratio where the ratio of the upper to lower 95% confidence intervals is >10).

Because we were still rating individual studies rather than a body of evidence, we excluded the fifth feature suggested by OHAT, publication bias. We summed the points for a maximum score of 8 and a minimum score of −4. Following OHAT protocol, any study that scored ≤0 was removed from the review at this point because we would have very low confidence in their outcomes (Office of Health Assessment and Translation, 2015). We were then able to assign the remaining studies a confidence rating: high, >5; moderate, 3–4; or low, 1–2. Thirty studies were independently reviewed by a second rater, and Cohen's kappa coefficient showed substantial agreement (0.67) (McHugh, 2012).

### Translate confidence ratings into evidence of desired behavior change (step 6)

We extracted data on the behavioral outcomes of each study, noting whether they resulted in positive behavior change, negative change, or no change. We then classified the level of evidence for desired behavior change that each study provided according to their confidence ratings and direction of effect (Figure 1, step 6). This strategy involved the use of three terms to describe the level of evidence for behavioral outcomes: *I*, *presumed*, and *suspected*, which were directly translated from the confidence‐in‐the‐evidence ratings. Because there were only four studies with negative or mixed results and they did not significantly affect our findings, we focused on evidence that a given variable would lead to positive or desired behavior change.

We were then able to collate the results from multiple studies to calculate the overall level of evidence for a given variable by calculating the mean of the numerical rating from different studies (from 3 to −3). If the mean confidence rating for a group of studies was <1, behavior change was suspected. If it was 1 ≥ X > 2, behavior change was presumed. If it was 2 ≥ X > 3, behavior change was known. These terms were taken from the OHAT protocol (Office of Health Assessment and Translation, 2015). We also sought to calculate an effect size for different intervention types. However, the number of papers that reported these or enough information to allow for their calculation was insufficient to allow for a meaningful analysis. Thus, we did not include effect sizes in this analysis.

### Data analyses

The OHAT protocol acknowledges that disparate exposure and outcome assessments may preclude formal statistical meta‐analysis and, therefore, does not specify statistical tests for outcomes (Office of Health Assessment and Translation, 2015). However, we conducted exploratory data analyses to further examine the relationships between variables. Our aim was to examine the effects of different variables on the distribution in the number of studies, variation in the quality of studies, and strength of evidence that a given intervention results in desired behavior change. Quality of studies refers to the confidence rating for each individual study, and the strength of evidence was based on the mean scores calculated for a body of studies as described in step 6. We were interested in whether there were differences in these outcomes across time, space, and thematic area. The type of intervention, the number of times audiences were exposed to the intervention, and the length of time for which the intervention ran were also potential variables of interest.

Data analyses were conducted in R 4.0.0, and we selected the appropriate statistical tests based on the dependent and independent variables. We employed a Kendall tau‐b correlation to test the distribution in the number of studies across time, and chi‐square tests were used to examine the distribution in the number of studies across all the other variables of interest (region, theme, intervention type, duration of intervention, and the number of exposures to interventions). We calculated a Spearman's rank correlation (rs) coefficient to test whether there was variation in the quality of studies across time and used a Kruskal–Wallis test to determine the distribution in quality of studies across the remaining variables of interest. We calculated Spearman's rank correlation coefficient to test whether the strength of evidence that a given intervention will result in desired behavior change varied across time, and again we used a Kruskal–Wallis test for the other variables. We also explored trends among the studies that were excluded from our review with Spearman's rank correlation coefficient to test whether the number and quality of published studies including a control varied over time.

Finally, we conducted a narrative synthesis to explore major themes and relationships between and within these studies in order to identify factors contributing to their reported success or failure (Popay et al., 2006).

## RESULTS

From initial identification of 338,408 records, we found 128 individual studies published in 107 articles that met our quality criteria for inclusion (Figure 2). Confidence ratings for these studies varied from low (*n* = 27), moderate (*n* = 55), and high (*n* = 48). Most studies reported a positive effect (*n* = 96), and the next most common outcome was no effect (28). Few studies reported negative (*n* = 1) or mixed (*n* = 3) effects. Unfortunately, only 25 (19%) of the 128 interventions reported enough detail in the statistical results to calculate a standardized effect size for meta‐analyses.

### Date of publication

Included studies were published from 1975 to 2015. The variation in the number of studies published over this period was not statistically significant (*z* = 1.36, *p* = 0.17). There was, however, a significant increase in the quality of studies (rs = 0.27, *p* = 0.002) and the strength of evidence that a given intervention would result in desired behavior change over time (rs = 0.21, *p* = 0.02).

### Location of intervention

**FIGURE 3:**
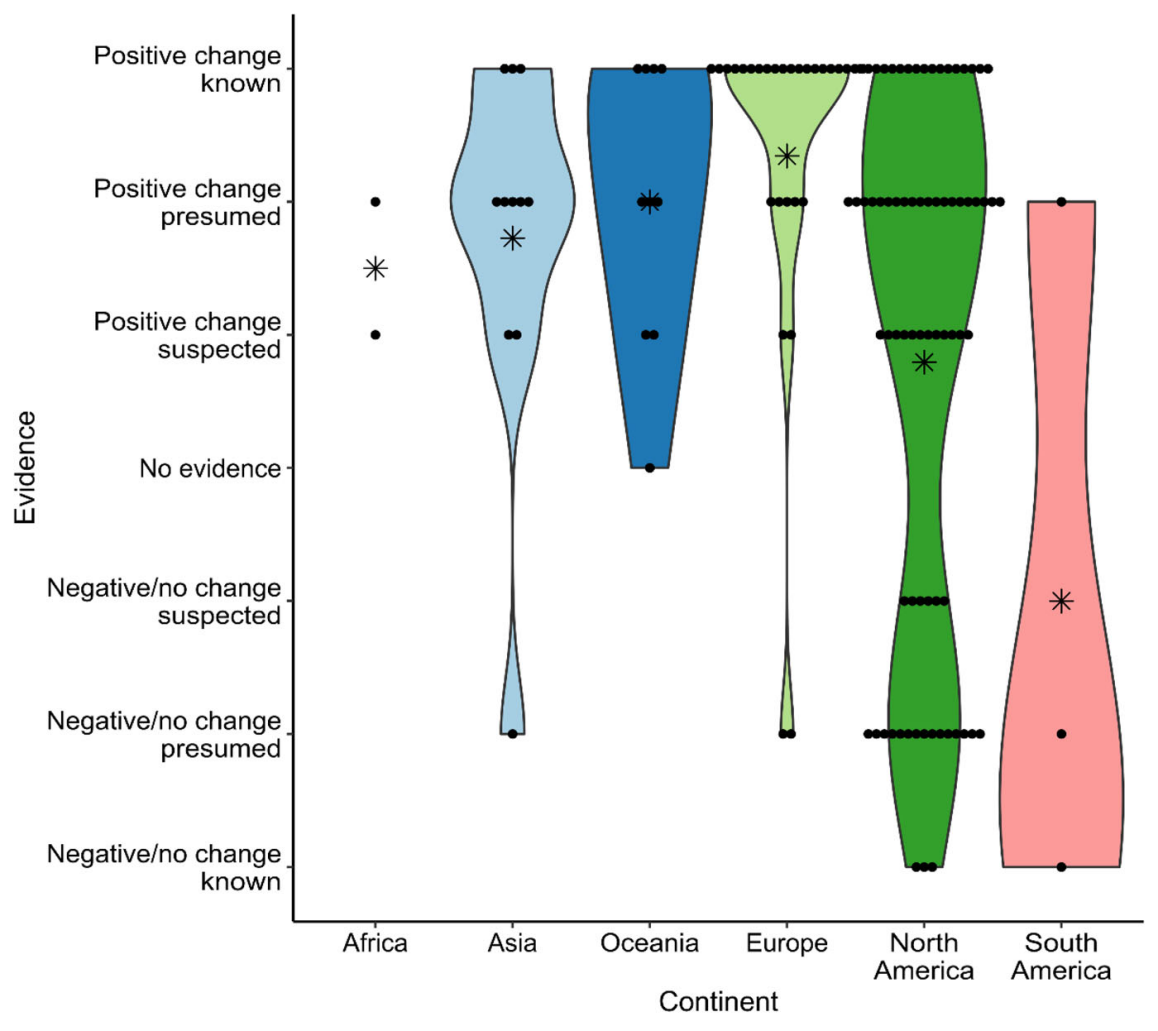
Strength of evidence that a nonpecuniary and nonregulatory intervention will lead to desired behavior change, by study location (points, individual studies; asterisks, mean rating; shading, violin plot)

There was an uneven distribution of studies across continents (χ^2^ = 172.19, df = 5, *p* < 0.001), and a disproportionate number was conducted in North America and Europe. The quality of studies across regions varied significantly (χ^2^ = 15.99, df = 5, *p* = 0.007); Europe featured an above‐average proportion of high‐quality studies. The level of evidence that a given intervention will result in desired behavior change also varied by study location (χ^2^ = 22.62, df = 5, *p* < 0.001). Those conducted in Asia, Europe, and Oceania were more likely to show strong evidence that intervention resulted in the desired behavior change (Figure 3).

### Study theme

Studies were unequally distributed across study themes (χ^2^ = 55.75, df = 5, *p* < 0.001), and a disproportionate number focused on waste and energy: agriculture and conservation (*n* = 5), energy (*n* = 40), transport (*n* = 17), waste (*n* = 41), water (*n* = 15), a mixture of these (*n* = 10). We could not reject the null hypothesis that there is no relationship between the quality of the studies and the study theme (χ^2^ = 3.57, df = 5, *p* = 0.61) or the null hypothesis that there is no relationship between the likelihood of a given intervention resulting in behavior change and the study theme (χ^2^ = 9.78, df = 5, *p* = 0.08).

### Intervention type

The distribution of studies was uneven across intervention types (χ^2^ = 65.92, df = 6, *p* < 0.001): conservation devices (*n* = 8), feedback (*n* = 24), goal setting (*n* = 10), prompt (*n* = 24), and a combination of the different types (*n* = 14). A disproportionate number focused on education interventions (*n* = 45) and very few examined demonstrations (*n* = 3). The combinations were feedback and goal setting (*n* = 4), education and conservation device (*n* = 3), feedback and education (*n* = 3), feedback, goal setting, and coaching (*n* = 1), education and demonstration (*n* = 1), prompt and conservation device (*n* = 1), and education, prompt, and demonstration (*n* = 1).

**FIGURE 4:**
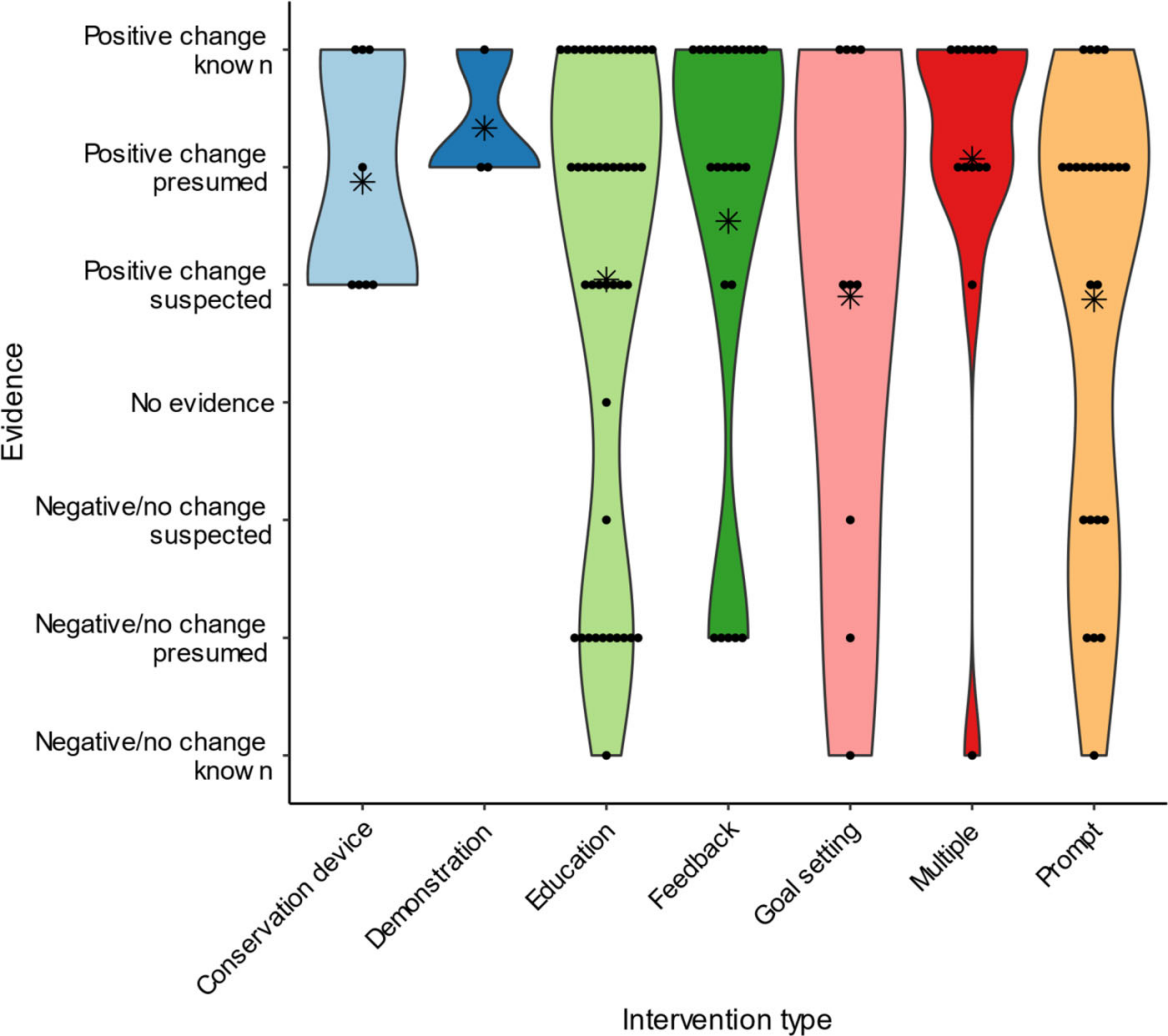
Strength of evidence that a given type of nonpecuniary and nonregulatory intervention will lead to desired behavior change (points, individual studies; asterisks, mean rating; shading, violin plot)

We could not reject the null hypothesis that there is no relationship between the quality of the studies in our review and the intervention type (χ^2^ = 7.74, df = 6, *p* = 0.26) or the null hypothesis that there is no relationship between the likelihood of a given intervention resulting in behavior change and the intervention type (χ^2^ = 6.86, df = 6, *p* = 0.33). However, we were able to assess the body of evidence for each type individually. Based on the quality and reported outcomes of studies examining interventions based on education, prompts, and feedback and their outcomes, we could presume that desirable behavior change will result from this intervention type (Figure 4). For goal setting, desirable behavior change is only suspected. We did not rate interventions based on conservation devices and demonstrations due to the low number of studies focusing on these intervention types. However, our highest confidence rests on the use of multiple different intervention types, for which positive change is a known outcome.

### Intervention duration and exposure frequency

**FIGURE 5:**
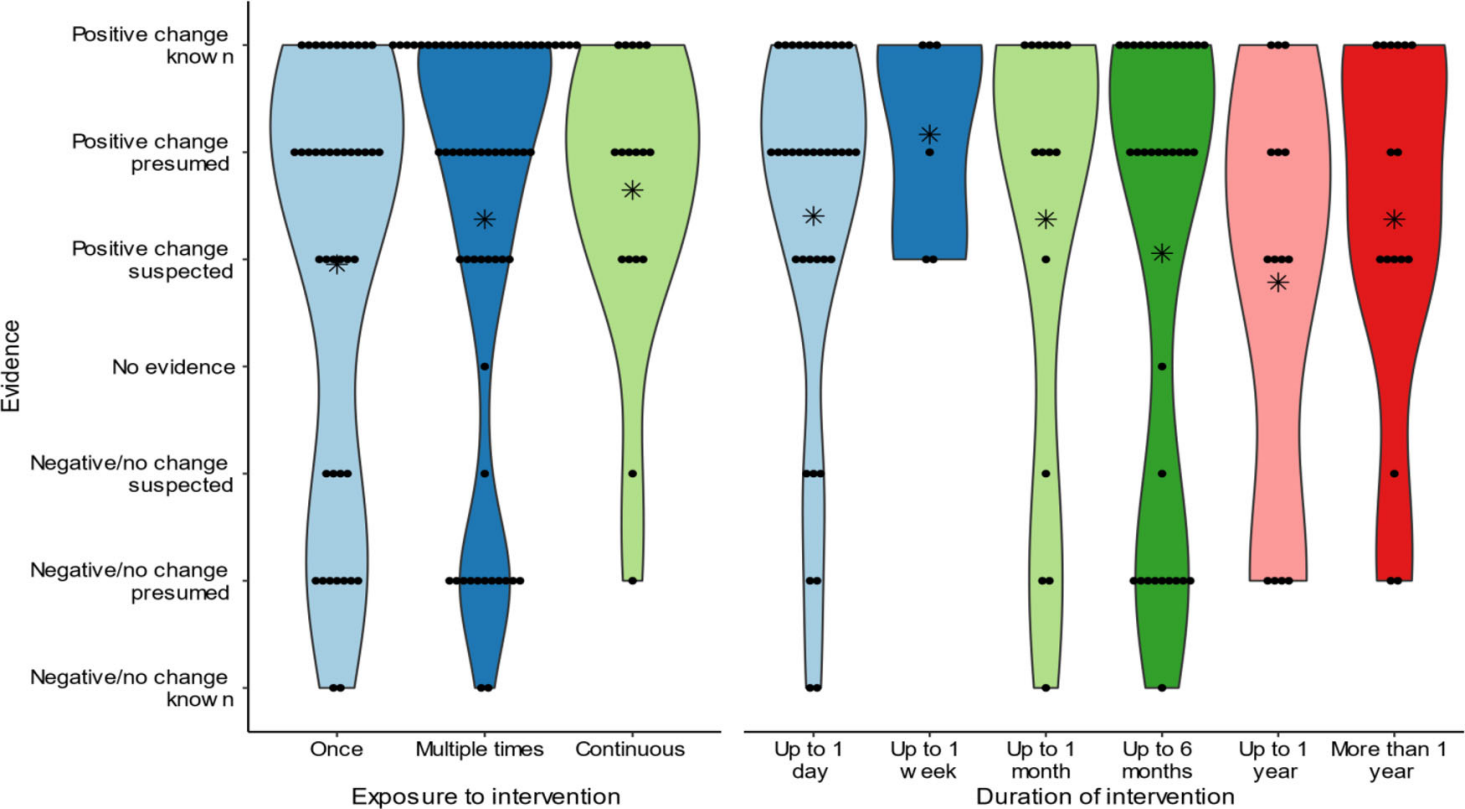
Strength of evidence that the type or duration of exposure to a nonpecuniary and nonregulatory intervention will lead to desired behavior change (points, individual studies; asterisks, mean rating; shading, violin plot)

We identified interventions that ran from 10 min to multiple years and audiences who may have been exposed to the intervention once, multiple times, or continually (e.g., a conservation device installed in the home). There was significant variation in the number of studies by both intervention length (χ^2^ = 37.52, df = 5, *p* < 0.001) and nature of exposure (χ^2^ = 26.82, df = 2, *p* < 0.001). There was a disproportionate number that lasted <1 day and 1–3 months and that involved multiple exposures. Study quality did not vary by intervention duration (χ^2^ = 9.2, df = 5, *p* = 0.1) or by the number of times an audience was exposed to an intervention (χ^2^ = 0.86, df = 2, *p* = 0.65). Overall, we could not reject the null hypotheses that there is no relationship between the likelihood of a given intervention resulting in behavior change and the quantity of exposures (χ^2^ = 1.78, df = 2, *p* = 0.41) (Figure 5) or duration of the intervention (χ^2^ = 8.18, df = 5, *p* = 0.15).

The number of studies published varied significantly across all variables of interest except the publication date (Table 4). The only variables for which we were able to detect a relationship with study quality and evidence for desired behavior change were the date of publication and location.

**TABLE 4:** Summary results for the impact of variables of interest on the number of studies published, quality of studies, and strength of evidence for desired behavior change

Variable	Number	Quality	Evidence
Publication date	*Z* = 1.36, *p =* 0.17	rs = 0.27, *p* < 0.01*	rs = 0.21, *p* = 0.02*
Location	χ^2^ = 172.19, df = 5, *p* < 0.01*	χ^2^ = 15.99, df = 5, *p* < 0.01*	χ^2^ = 22.62, df = 5, *p* < 0.01*
Thematic area	χ^2^ = 55.75, df = 5, *p* < 0.01*	χ^2^ = 3.57, df = 5, *p* = 0.61	χ^2^ = 9.78, df = 5, *p* = 0.08*
Intervention type	χ^2^ = 65.92, df = 6, *p* < 0.01*	χ^2^ = 7.74, df = 6, *p* = 0.26	χ^2^ = 6.86, df = 6, *p* = 0.33
Intervention length	χ^2^ = 37.52, df = 5, *p* < 0.01*	χ^2^ = 9.2, df = 5, *p* = 0.1	χ^2^ = 8.18, df = 5, *p* = 0.15
Number of exposures	χ^2^ = 26.82, df = 2, *p* < 0.01*	χ^2^ = 0.86, df = 2, *p* = 0.65	χ^2^ = 1.78, df = 2, *p* = 0.41

* *p*<0.05.

### Ineligible studies

For studies that we excluded from our review, the publication of studies including controls decreased over time (rs = −0.19, *p* = 0.03). However, in the subset that did include a control, quality improved (rs = 0.23 *p* = 0.01). We could not reject the null hypothesis that there is no relationship between the presence of control (χ^2^ = 0.31, df = 1, *p* = 0.58) or quality (χ^2^ = 5.51, df = 5, *p* = 0.36) and studies in the gray versus peer‐reviewed literature.

## DISCUSSION

We found strong evidence that education, prompts, and feedback interventions can result in positive behavior change. Based on our results, combining multiple interventions in one campaign is most likely to lead to the desired outcomes. However, we could not determine what combinations of intervention types worked best for what behaviors, in what contexts, and for what duration and intensity. Surprisingly, we were unable to reject the null hypothesis that there is no relationship between the overall duration or frequency of exposure to the intervention and the likelihood of positive behavior change. However, the quality of the studies and the strength of the evidence overall varied by date of publication and location of study. This may be due to greater resources available to researchers in the West (Waldron et al., 2013).

Geographic location was the only variable that significantly affected the distribution, quality, and evidence strength of studies. This continental bias toward North America and Europe has unfortunately also been identified in other reviews of conservation research and limited our ability to draw firm conclusions about interventions’ effectiveness beyond these locations (Wilson et al., 2016). Previous research in behavioral science shows that there is substantial variability in experimental results across populations, and a lack of cultural diversity in research participants could skew responses to interventions (Henrich et al., 2010).

Results from our systematic review showed an average of three high‐quality studies published each year globally. At the same time, nearly two‐thirds of eligible studies (223) had to be removed from our review because they failed to include an independent control. A rigorous impact evaluation should provide credible evidence by using an appropriate counterfactual to establish causal attribution (Ferraro, 2009). Control groups act as a counterfactual, thereby mitigating bias in the comparison of impacts when bias in the allocation has been taken into account. Less than 20% of included studies presented enough statistical information to determine effect size. Unfortunately, the need to incorporate better‐designed measurement protocols in environmental‐behavior‐change interventions is another conclusion that repeatedly arises in reviews of the literature (Byerly et al., 2018; Delmas et al., 2013). Byerly et al. (2018) recently noted that many studies are poorly designed, lacking adequate controls and sufficient statistical power. Considering the exponential spread of anthropogenic threats to the environment and the urgency for effective mitigation strategies, it is vital to improve the rigor with which impact evaluation is approached and pay this issue the attention it deserves (Cowling, 2014; Fischer et al., 2012). This widespread and systemic failure should be a call to action for all conservation social scientists.

### Narrative syntheses

In our narrative synthesis, the cohort of studies spanned 40 years, and more recent studies built on findings from earlier benchmark studies. For example, Carrico's (2009) intervention design, focusing on motivational feedback, was informed by Becker's (1978) findings that goal commitment without feedback is ineffective. Further, most of the studies were informed by the large body of current behavioral theories in the social sciences, such as the nudge theory (Baca‐Motes, 2013, Baird, 2013), moral norms (Ayres et al., 2009; Thφgersen, 1999), theory of planned behavior (Thøgersen, 2009), habit hypothesis (Bamberg 2006), and motivational feedback (Becker 1978).

The design of intervention seemed strongly related to the problem it was intended to address. For example, researchers working on energy consumption tended to employ multifaceted interventions, including the provision of educational material (Carrico 2009), individual consumption feedback (Hayes 1977), and peer feedback and goal setting (Loock, 2013), whereas those focused on litter prevention and waste management tested more visual interventions, such as signage and prompts (Sussman, 2013, Hansmann 2003).

The exposure of the targeted audience to the interventions varied widely, ranging from a one‐off exposure event to months or even years. The number of times the intervention was implemented also varied. There appears to be little consensus on appropriate exposure times for eliciting a behavioral response. Several studies noted the importance of continuous follow‐up or lack of follow‐up and lengthening exposure time to increase the likelihood of a successful outcome (Baca‐Motes 2013; Baird 2013; Harrigan 1994; Harrigan 1994; Hayes 1977), yet few studies included long‐term monitoring of behaviors. When researchers did conduct long‐term monitoring, the initial reported positive behavioral changes often diminished over time, perhaps suggesting a need for consistency in interventions over a longer period to achieve the desired long‐term behavioral change. This diminished impact over time occurred despite the likely attrition of participants who ceased the behavior change compared with those who maintained the desired behavior change. Unfortunately, the variability between studies, such as the length of the intervention relative to the follow‐up duration, limited our ability to suggest a best practice time frame for future research.

Rarely, outcomes besides the intended proenvironmental behavior were noted. Unintended detrimental effects following interventions were cited in several studies. For example, in one case, increased energy usage occurred after receipt of peer feedback (Ayers 2012). Different reasons were put forward to explain such undesirable outcomes, including the “boomerang effect” and “moral licensing” (Ayres 2012, Nomura 2011). Tiefenbeck (2013) recorded moral licensing when participants in a water conservation campaign reduced their water consumption as intended, but increased their electricity use.

Unintended or additional outcomes can also be beneficial, such as in the case of positive spillover (Evans 2013; Haq 2008). For example, environmental messages that promote car sharing for reasons other than personal benefit may also lead to an uptake in recycling (Evans 2013). However, cross‐domain adoption of additional proenvironmental behaviors as a byproduct of interventions was not commonly measured (*n* = 6).

One common theme that emerged was that studies documenting positive change tended to include behavioral strategies that made personal connections between the broader issues and the target audience. For example, researchers elicited emotional responses; building empathy or aligning with an individual's internal standards (Hansmann 2003). Strategies that lead to an emotional reaction can result in a positive behavioral change (Sussman, 2013). These approaches result in individual‐led action, linked to high‐motivation methods and making cognitive connections. This suggests that if an intervention is thought‐provoking and connects with audiences on a higher cognitive level, it is more likely to result in positive behavior change (Hansmann 2003; Miller 2009).

### Comparison with other reviews

During the initial literature search, we tried to be as inclusive as possible. We used broad search terms and searched in multiple bibliographic databases and languages (Haddaway & Macura, 2018). We also included a wide breadth of journals from different disciplines, although a more formal effort to benchmark search comprehensiveness with a set of papers already identified within a category would have allowed us to better understand the degree to which we were capturing all the relevant literature. What distinguishes our review from previous reviews is the broad screening criteria for experimental design and subject focus and the thorough critical appraisal process we used to classify all the studies that met inclusion criteria. Instead of vote counting based on the statistical significance or the exclusive on one kind of experimental design, such as randomized controlled trials, we weighted the studies by quality to measure the strength of evidence (Haddaway & Macura, 2018). In addition, any study that did not meet a certain threshold for rigor was removed from the final analysis. This means that all the studies in our review still met key standards of robustness, which is important because previous reviews show that behavioral effect sizes vary with study rigor (Delmas et al., 2013). We also included a wide range of gray literature, which is often overlooked in other reviews due to concerns about study quality. However, we found no significant differences in quality between the gray and peer‐reviewed literature.

Results of previous reviews suggest that although single policy tools frequently fail to reduce household energy consumption, synergistic effects can come from combining interventions (Dietz et al., 2009). For example, the most effective interventions for daily energy‐use behaviors generally involve a mixture of mass‐media messages, household‐specific information, and social influences. Indeed, combining feedback with goal setting works particularly well in the energy sector (Abrahamse et al., 2005). This echoes a notable finding from our review: we found strong evidence to support the effectiveness of multiple interventions, such as feedback plus goal setting and education plus conservation devices.

Previous reviews of behavior change interventions in, for example, public health suggest that intervention success may be linked to intensity. The weight of evidence shows that long campaigns with frequent contact time lead to positive outcomes, such as greater weight loss or a reduction in risky sexual behaviors (Chandra‐Mouli et al., 2015; Greenhow, 2011; Robin et al., 2004). However, interventions often vary considerably in duration and delivery, preventing even a descriptive analysis, let alone the identification of an optimal formula (Durlak & DuPre, 2008; McCoy et al., 2010; Wei et al., 2011). We found no clear link between intervention success and duration of exposure, but there may be heterogeneity among different intervention types. This should be a priority area for future research.

### Methodological considerations

As with all syntheses, there is the possibility that the studies we identified were subject to the “file‐drawer effect” or were biased toward the publication of studies with positive and significant results (Franco et al., 2014; Scargle, 2000). For example, publication bias has been shown in previous reviews of behavioral science literature (Francis, 2012). A visual assessment of outcome distributions (number of studies published with positive rather than negative or no change) certainly suggests that publication bias may be a concern in Asia, Africa, Europe, and Oceania, but less so in North and South America (Figure 3). Further, the absence of published studies showing negative results for intervention types, such as conservation devices or demonstrations, is alarming, and limits what we can say about the true effectiveness of these interventions.

Research from the medical field shows that when studies are preregistered, negative outcomes are more likely (Dwan et al., 2008). Moves by journals, such as *Conservation Biology* to allow preregistration, are a step in the right direction to address publication bias. For this review, we tried to mitigate publication bias by searching both the peer‐reviewed and gray literature (Haddaway & Macura, 2018). We also included a wide breadth of journals from different disciplines. Our focus on direct behavioral outcomes likely restricted eligible studies to topics where behavior can feasibly be measured. This does not mean that interventions have been ineffective in changing more elusive behaviors, such as the consumption of illegal wildlife trade products, but rather there is not yet enough evidence to come to a confident conclusion (Veríssimo & Wan, 2018). In addition, grouping diverse behaviors, from water use to transport choices, may have masked interesting trends in the relative effectiveness of different interventions (Heimlich & Ardoin, 2008). Currently, however, the lack of studies prevents a more detailed analysis of these possible interactions.

Since 2015, when we conducted our search, multiple high‐quality, rigorous studies have been published that would have met the criteria for our review (Schwartz et al., 2020; Weigel et al., 2021; Wolstenholme et al., 2020). It is possible that including these studies would have improved the average robustness of the research featured in this review. However, it is also worth noting that the latest literature on the use of behavioral science to conserve biodiversity continues to identify most, if not all, of the challenges we highlighted above, including lack of controls, narrow geographic focus, and failure to measure actual behaviors (Balmford et al., 2021; Nilsson et al., 2020; Palm‐Forster et al., 2019).

### Summary and future work

Several key gaps in the literature need to be addressed. Comparatively few studies tested the effects of voluntary interventions on non‐Western populations or measured actual conservation behaviors. Although prompts and education are well‐studied, evidence to support the use of conservation devices and demonstrations is lacking. Future researchers should aim to fill these gaps and should improve reporting standards. More detail is needed both on the statistical front to enable the calculation of effect sizes and in terms of intervention implementation. For example, we had to use a coarse categorization scheme for the duration and frequency of exposure analyses. If we had the quantity of information for more fine‐grained analysis, our results would have been more robust. Finally, the extent to which behavior change persists after the intervention has ceased needs investigation (Burns & Savan, 2018; Byerly et al., 2018).

We found strong evidence that a range of different, well‐designed intervention types can result in desired behavior change. The strongest evidence came from the combination of multiple intervention types, for example, both conservation devices and education. Encouragingly, we found successful interventions across a range of durations and exposures, indicating that behavior change can occur from even short‐term efforts. This does not mean the role of governments and industry in addressing major environmental issues should be downplayed; instead, it highlights some of the effective approaches they can use to maximize impact. The findings from our review should be used by practitioners to guide future interventions and by researchers to inform future studies.

## Supporting information

Appendix S2. Databases were searched using ISI Web of Science and EBSCOhost
Appendix S3. Records retrieved per database/platform

Supporting Material

Supporting Material
