# Supplementary material for: Systematic review of conservation interventions to promote voluntary behavior change
Source: Conserv Biol. 2022 Dec 8;37(1):e14000. doi: 10.1111/cobi.14000 (PMC10108067; doi:10.1111/cobi.14000)
Supplement: Supplementary file 1 — Appendix S2. Databases were searched using ISI Web of Science and EBSCOhost Appendix S3. Records retrieved per database/platform [file COBI-37-0-s002.docx]

**Appendix 2. Databases searched using ISI Web of Science and EBSCOhost**

**Web of Science: (all available)**

- Web of Science Core Collection
- BIOSIS Citation Index
- Chinese Science Citation Database
- Russian Science Citation Index
- SciELO Citation Index
- Biological Abstracts, BIOSIS Previews
- CABI: CAB Abstracts and Global Health
- KCI—Korean Journal Database
- Medline
- Zoological Record

**EBSCOhost:**

- Academic Search Complete
- Business Source Complete
- Child Development & Adolescent Studies
- Communication & Mass Media Complete
- Consumer Health Complete - EBSCOhost
- EconLit
- Education Source
- Environment Complete
- ERIC
- Family & Society Studies Worldwide
- Global Health
- GreenFILE
- Health Source - Consumer Edition
- Hospitality & Tourism Complete
- Information Science & Technology Abstracts (ISTA)
- MedicLatina
- MEDLINE
- MEDLINE with Full Text
- Newspaper Source
- Newswires
- Professional Development Collection
- PsycARTICLES
- PsycEXTRA
- Psychology and Behavioral Sciences Collection

**Appendix 3. Records retrieved per database/platform**

| ISI Web of Science | 72359 |
| --- | --- |
| SciVerse’s Scopus | 31500 |
| EBSCOhost | 99183 |
| International Bibliography of the Social Sciences | 5821 |
| Google Scholar | 25687 |
| ProQuest Digital Dissertations and Theses | 65493 |
| PolicyFile | 281 |
| My Environmental Education Resource Assistant | 40 |
| Canadian Evaluation Society Unpublished Literature Bank | 1 |
| System for Information on Grey Literature in Europe | 3314 |
| CORDIS Library | 86919 |
